# Prevalence of Surgical Glove Perforation in Orthopedic Surgery at Tribhuvan University Teaching Hospital: An Observational Study

**DOI:** 10.31729/jnma.8931

**Published:** 2025-04-30

**Authors:** Prawesh Singh Bhandari, Shirish Adhikari, Nitish Bikram Deo, Suresh Uprety, Priska Bastola

**Affiliations:** 1 Department of Orthopaedics and Trauma Surgery, MMC, Kathmandu, Nepal; 2 Department of Cardiothoracic and Vascular Anaesthesiology, Manmohan cardiothoracic and vascular centre, MMC, Kathmandu, Nepal

**Keywords:** *occult*, *orthopedic surgery*, *perforation*, *surgical glove*, *trauma*

## Abstract

**Introduction:**

Gloves provide a physical barrier preventing cross-infection between the operating team members and the patient. However, there is always a chance of the gloves perforating and breaching this barrier. This study attempts to understand the prevalence of perforation of surgical gloves in orthopedic surgery at Tribhuvan University Teaching Hospital.

**Methods:**

This was an observational cross-sectional study carried out over three months. Gloves from the chief and first assistant surgeons were checked for visible perforation and occult perforation by water leak test. The type of surgery, hand dominance, duration of surgery, time during surgery when perforation occurred, and area of glove perforation were noted. Operative perforation rate, Overall glove perforation, and Operative perforation based on type of surgery, and duration of surgery were calculated. The categorical variables obtained were summarized with frequency and percentage.

**Results:**

A total of 166 cases were included in the study. The operative perforation rate was 79 (47.59%; 95% CI: 39.80-55.47%) and the overall glove perforation was 117 (8.81%). Trauma surgery was the most common surgery performed during this study 111 (66.86%) and 56 (50.45%) of trauma surgery cases had glove perforation. Glove perforation was noticed by 25 (43.85%) of chief surgeon during surgery, out of which 11 (44%) of it was between 0.5 to 1 hour. Similarly, 20 (50%) of assistant surgeon noticed glove perforation during surgery, out of which 9 (45%) of it was between 1 to 1.5 hour after starting the surgery.

**Conclusions:**

Perforations of the surgical gloves was comparable to other published literature. Trauma surgery was the most common.

## INTRODUCTION

Surgical gloves provide an effective physical barrier between surgeons and the patient's body protecting the surgeons from acquiring the blood-borne infectious disease and the patients from surgical site infection (SSI).^1,2^ These effective barriers are often breached during surgery.^1,2^ Glove perforations occur at different rates among different surgical specialties and also vary among various sub-specialties of orthopedic surgery.^3^ Orthopedic surgeons are especially vulnerable to this phenomenon as orthopedic surgeries include cutting, drilling, use of pointed instruments and implants, and handling of sharp bone fragments.^4^

There is no study about the prevalence of surgical glove perforations among orthopaedic surgeries. This information will provide an insight so as to plan for the complication preventions. Therefore, this study aims to find out the prevalence of surgical glove perforation among orthopaedic surgeons and also to note the time at which perforations occur, the type of perforations (visible or occult), and the area of the glove where perforations occur.

## METHODS

This is an observational cross-section study conducted at Orthopedic operation theatre in Tribhuvan University Teaching Hospital (TUTH) over 3 months from 1st June 2021 to 30th August 2021. The study was conducted after obtaining ethical approval from the Institutional Review Board, Institute of Medicine (Reference number: ??). The sample size was calculated using a 12% proportion of glove perforation from a previous study, with a 5% margin of error at a 95% confidence interval (CI). The sample included 166 elective and emergency orthopedic surgeries — including spine, arthroplasty, arthroscopy, pediatric, hand, infection, oncology, and trauma surgeries — selected conveniently, where surgical glove perforations were observed.

Cases requiring multispecialty surgery in the same setting and cases undergoing surgery under local anesthesia were excluded from the study.

Study variables included type of orthopedic surgery (Trauma Surgery, Spine Surgery, Paediatric orthopedic surgery, Arthroplasty, Arthroscopic surgery, and Others), duration of surgery (< 1 hour, 1-2 hours and >2 hours), glove perforation if present or absent and the number of perforated gloves. For both chief and assistant surgeons, hand dominance (Right or Left), time of perforation (during the surgery or end of surgery), and area of perforations of both inner and outer right and left gloves (index finger, thumb, palm, and others) were noted. The time of perforation was noticed by surgeon and recorded as <0.5, 0.5 to 1, 1 to 1.5, 1.5-2 and >12hour) was noted. Perforations noted during the surgeries were taken as visible perforations. Perforations noted at the end of the surgery after collection by the investigator were classified (visible and occult) and recorded. Visible perforations were noticed by the naked eyes of the investigator while the occult perforations were only noticed after subjecting each individual glove to the Water Leak Test (WLT).^6^ The WLT method (EN 455-1), has been approved by the European and normalization committee as well as Food and Drugs Administration (FDA). Each glove was filled with 1000 ml ±50 ml of water at room temperature and gently squeezed to find out for the perforation. Case of a positive perforation of the surgical glove was defined as the presence of droplets or stream of water on the exterior aspect of the surgical glove. The gloves with water leaking out on water insufflation were reported as occult perforations. The type of perforation and area of glove perforated were noted for each of the collected gloves separately. The principal investigator collected both inner and outer gloves from both the surgeon and the assistant surgeon and evaluated for any perforation. The latex pre-powdered gloves used for evaluation were procured by the hospital.

Data were collected in the predefined data collection tool and entered and analysed in Microsoft Excel (Microsoft Office Professional Plus 2019). All the variables were categorical, and descriptive statistics were used and measures of outcomes were represented as frequency, percentage, and proportions. The operative perforation rate is calculated as the number of cases with glove perforation out of the total cases. Overall glove perforation is defined as the total number of gloves perforated to the total number of gloves worn. In all our surgeries both chief and assistant surgeons used both outer and inner gloves in both hands.

## RESULTS

A total of 166 surgeries were observed for glove perforation out of which 79 (47.59%; 95% CI: 39.80-55.47%) surgeries had the incident of glove perforation. There were four pair (eight) gloves used in each surgery, therefore a total of 1328 gloves were used. Out of the total gloves used 117 (8.81%) were perforated. There were 56 (50.45%) glove perforation during trauma surgery and 23 (57.50%) in the surgeries lasting more than two-hour, (Table 1 and 2).

**Table 1:** Proportion of gloves perforation in various orthopedics surgery (n=166).

Type of Surgery	Cases n (%)	Operative Perforation n (%)
Arthroscopic Surgery	12 (7.22)	5 (41.66)
Arthroplasty	5 (3.01)	4 (80)
Trauma Surgery	111 (66.86)	56 (50.45)
Spine Surgery	26 (15.66)	12 (46.15)
Pediatric Orthopedic Surgery	4 (2.40)	-
Others	8 (4.81)	2 (25)

**Table 2:** Proportion of glove perforation in surgeries of various time duration (n=166).

Operative Duration	Cases n (%)	Operative Perforation n (%)
< 1 Hour	52 (31.32)	17 (32.69)
1-2 Hours	74 (44.57)	39 (52.70)
> 2 Hours	40 (24.09)	23 (57.50)

**Table 3:** Time-period of glove perforation during orthopedic surgery (n=166).

**Time When Perforation Was Noted for Cheif Surgeon**	
At End Of Surgery	32 (56.14)
During the surgery	25 (43.85)
**Time When Perforation Was Noted (Hours) for Cheif Surgeon**	
< 0.5	3 (12)
0.5 - 1	11 (44)
1 - 1.5	10 (40)
1.5 - 2	-
> 2	1 (4)
**Time When Perforation Was Noted for Assistant Surgeon**	
At End Of Surgery	20 (50)
During the surgery	20 (50)
**Time When Perforation Was Noted (Hours) for Assistant Surgeon**	
< 0.5	-
0.5 - 1	5 (25)
1 - 1.5	9 (45)
1.5 - 2	-
> 2	6 (30)

Glove perforation was noticed by 25 (43.85%) of chief surgeon during surgery, out of which 11 (44%) of it was between 0.5 to 1 hour. Similarly, 20 (50%) of assistant surgeon noticed glove perforation during surgery, out of which 9 (45%) of it was between 1 to 1.5 hour after starting the surgery (Table 3).

There were 27 (84.37%) occult perforation in chief surgeon's and 16 (80%) in assistant surgeon's gloves. In case of chief surgeon perforation with instrument was 14 (43.75%) and it was 9 (45%) for assistant surgeon. The outer glove perforation in index finger of chief surgeon was 23 (40.35%) and 15 (37.50%) for assistant surgeon (Table 4).

**Table 4:** Proportion of glove perforation in surgeries of various time duration (n=166).

**Type of Perforation at the End of Surgery for Chief Surgeon**		
Occult	27 (84.37)	
Visible	5 (15.62)	
**Type of Perforation at the End of Surgery for**		
Assistant Surgeon		
Occult	16 (80)	
Visible	4 (20)	
**Area of Glove Perforation for Cheif Surgeon**		
**Chief Surgeon**	**Inner n (%)**	**Outer n (%)**
**Left Hand**		
Index	4 (1.75)	23 (10.09)
Thumb	1 (0.44)	11 (4.82)
Palm	1 (0.44)	2 (0.88)
Others	1 (0.44)	3 (1.32)
**Right Hand**		
Index	4 (1.75)	12 (5.26)
Thumb	-	6 (2.63)
Palm	-	4 (1.75)
Others	1 (0.44)	4 (1.75)
**Assistant Surgeon**		
**Left Hand**		
Index	3 (1.88)	15 (9.38)
Thumb	1 (0.63)	4 (2.50)
Palm	-	1 (0.63)
Others	-	4 (2.50)
**Right Hand**		
Index	-	6 (3.75)
Thumb	1 (0.63)	5 (3.13)
Palm	1 (0.63)	3 (1.88)
Others	-	2 (1.25)
**Chief Surgeon's Hand Dominance**		
Left	2 (1.20)	
Right	164 (98.79)	
**Assistant Surgeon's Hand Dominance**		
Left	7 (4.21)	
Right	159 (95.78)	
**Cause of Perforation of Cheif Surgeon**		
K-Wire	4 (12.5)	
Instruments	14 (43.75)	
Suture Needle	1 (3.12)	
Sharp Bone	2 (6.25)	
Unknown	4 (12.5)	
**Cause of Perforation of Assistant Surgeon**		
K-Wire	1 (5)	
Instruments	14 (43.75)	
Suture Needle	2 (10)	
Sharp Bone	2 (10)	
Unknown	6 (30)	

## DISCUSSION

In our study operative perforation rate of gloves was 79 (47.59%) and overall peforation rate was 117 (8.81%), this was comparable to a study where the operative perforation rate was 49% and the overall glove perforation rate was 16%.^7^ In a similar study done in arthroplasty and arthroscopic orthopedic surgery in 200 cases in Turkey, the overall perforation rate was 15.8% and the operative perforation rate was 35%.^8^ In a study done on primary Total knee arthroplasty in South Korea in 2011, the overall perforation rate was 4.3% while the operative perforation rate was 13.6%.^9^ In a study by Choudhary & Padia (2015), the perforation rate of gloves in overall orthopedic surgery was found to be 12.6% with an operative perforation rate of 45%.^10^ In another study, Joseph et al. (2018) found that the overall perforation was 17.1% and higher perforations were seen in bone surgeries.^3^ Dhar (2011) in their study conducted in cases of primary and revision total hip arthroplasty found that the outer glove perforation rate was 38.33% and the inner glove perforation rate was 25%.^11^ In another study, glove perforation among different orthopedic subspecialties like pediatric, hand, and spine was compared, which showed an overall glove perforation rate of 8.7% (69/792) and an operative perforation rate of 45% (45/100). The hand operations had the lowest operative perforation rate (19.4%) while the spine operations had the highest (63.6%). The glove perforation rate increased in bony procedures (60% versus 22.5%), and procedures with major instrumentation (66% versus 18%).^6^ In the case of spine surgery, the overall operative perforation rate was 51.4%.^4^ The above results of various studies suggest that even though there is a difference in reporting, the results follow a similar pattern where perforations are more common in cases of surgeries involving the handling of bone and instruments. In our study, the overall prevalence might have been high due to a greater percentage of bone surgery, especially trauma surgery. The increased prevalence of chief surgeon glove perforations may be due to the active manipulation of bone and tissues with hands and instruments. The result of our study goes on to show that the prevalence of glove perforation in our setting is comparable to other studies. Our findings underscore the importance of increased vigilance on glove perforations among orthopedic surgeons, particularly, when the surgeries involve extensive bone handling. This also supports our practice of using double gloves.

In this study Glove perforation was noticed by 25 (43.85%) of chief surgeon during surgery, out of which 11 (44%) of it was between 0.5 to 1 hour. Ersozlu et al also found a lower incidence (25.2%) of glove perforations in chief surgeons as compared to assistant surgeons (8.3%).^7^ Comparable findings have been reported in earlier studies as well. In a study by Sanaullah et al., (2014), that included a total of 85 lower-limb trauma surgeries, the operative perforation rate was 49% and the overall glove perforation rate was 16%.^8^

In our study, most of the glove perforations occurred during the first two hours of the surgery since the majority of the surgeries lasted for that duration. The rate increased with the duration of the operation. In a previous study, in those cases where operations were shorter than 1 hour, 1.4 gloves per operation perforated as compared with 3.7 gloves per operation in operations longer than 2.5 hours.^12^ In a study by Joseph. K et al. (2020), surgeries lasting longer than 90 minutes were significantly associated with surgical gloves perforation.^3^ The perforations may be the consequence of fatigue due to prolonged use. As the duration of surgery increased so did the rate of perforation. Thus surgeons need to be more vigilant about the integrity of the glove as the duration of surgery gets longer. Hourly vigilance and change of gloves during bony procedures by the surgeons may be helpful.

Our study found that both chief and assistant surgeons missed perforation during the surgery but most of those missed perforations were occult. In a study that investigated the frequency of unnoticed surgical glove perforation at 3 different Tunisian university hospitals in 4 different surgical specialized units, it was found that the glove perforation rate was 22.5%.^13^ The results reported by Tlili et al. (2018) were comparable with a rate of 16.5%.(14) Ersozlu et al reported 72.7% of undetected occult perforation which is higher than any of the other studies.^7^ In a study by Han et al nearly 68% of the perforations were missed during the surgery.^9^ These studies didn't include a uniform group of surgeries and most cases were non-orthopedic surgeries which might be the cause of the difference. A significant number of perforations can go undetected to visual inspection which in our study were detected by water insufflation and checking for leaks thus further emphasizing the need for regular glove changes during prolonged and bony surgeries.

Our study showed that in both the assistant and chief surgeons, the index finger of the left hand was the part of the surgical glove most prone to perforation. Laine et al. (2001) reported a left-hand perforation rate of 35.8% (67) followed by a left-thumb perforation rate of 18.80%.(15) Right thumb perforation (29.3%) followed by left index finger (23.5%) was the common pattern in a study done by Ersozlu et al.^7^ In another study, the most common site of glove perforation was the index finger (47%).^5^ Han et al also found the index finger to be the most common site of perforation followed by the thumb.^9^ The most common location of surgical glove perforation was found to be the index finger at 36.9% in the study by Joseph K et al.^3^ All these results consistently point out that the gloves at the index finger of the non-dominant hand are most commonly perforated. Most of our surgeons were right-hand dominant and since the non-dominant hand is less dexterous of the two and is used in the prolonged activity of holding bone fragments during reduction, palpation, and manipulation while the more dexterous right hand is used to manipulate instruments, the chances of injury to the index finger and thumb of the left hand is higher. This might explain the reason for left-hand index finger perforation occurring commonly. As shown by the study of Han et al thick latex gloves did not provide better protection as compared to conventional gloves.^9^

We feel that there is a paucity of literature in our part of the world regarding the issue of glove perforation and its implications. Thus the findings of this study can serve as a scientific basis to develop safety guidelines for the surgical team regarding the proper usage of the surgical glove and pave a path for further extensive and multicenter research. The study had few limitations. It was a single-center study and the researcher had no control over the quality of the gloves procured by the hospital.

## CONCLUSIONS

Surgical glove perforations are comparable with other published studies. Trauma surgery was the most common type of surgery leading to glove perforation and most of which were detected at the end of the surgery. Similarly, the outer glove and index finger was the common site for glove perforation both for chief surgeon and assistant surgeon.
